# Reply to: “Interpreting infection–disease associations in psoriasis and atopic dermatitis”

**DOI:** 10.1016/j.jdin.2026.06.013

**Published:** 2026-06-25

**Authors:** Zhiru Zhou, Yi Xiao

**Affiliations:** a Department of Dermatology, Xiangya Hospital, Central South University, Changsha, Hunan, P.R. China; b Hunan Engineering Research Center of Skin Health and Disease, Xiangya Hospital, Central South University, Changsha, Hunan, P.R. China; c Hunan Key Laboratory of Skin Cancer and Psoriasis, Xiangya Hospital, Central South University, Changsha, Hunan, P.R. China; d National Clinical Research Center for Geriatric Disorders (Xiangya Hospital), Central South University, Changsha, Hunan, P.R. China

**Keywords:** atopic dermatitis, disease phenotype, infection, meta-analysis, psoriasis, temporality

*To the Editor:* We thank Islam et al for their valuable insights regarding our recent meta-analysis.^1^ We agree that temporality, disease phenotype, infection subtype, and treatment exposure are important considerations when interpreting associations between infection and the subsequent development of psoriasis and atopic dermatitis (AD). Their comments provide an opportunity to further clarify the interpretation of our findings.

Regarding temporality, we acknowledge that reverse causation is a recognized limitation in observational research. Importantly, our meta-analysis was restricted to cohort studies in which infection exposure preceded disease diagnosis, which represents a methodological strength over cross-sectional designs. We further acknowledge that infections occurring shortly before diagnosis may reflect prodromal disease activity, impaired barrier function, increased healthcare utilization, or other disease-related factors rather than purely upstream causal exposure. Although some included studies attempted to address this issue by excluding events occurring shortly after infection, such approaches were not consistently applied across the literature.^2^^,^^3^ As discussed in our limitations, we agree that findings should be interpreted with appropriate caution regarding causal inference.

We acknowledge that detailed phenotype information was unavailable in most studies. However, our meta-analysis was designed to estimate population-level infection–disease associations in real-world settings, which is a distinct and complementary objective to mechanistic studies focused on specific phenotypes such as guttate psoriasis. To partially address the concern regarding infection specificity, we conducted subgroup analyses stratified by pathogen class and anatomical site of infection, and provided individual study-level infection categories and disease phenotypes in Supplementary Table I, available via Mendeley at https://data.mendeley.com/datasets/b5fvbd9djz/1. We agree that future studies with phenotype-stratified designs will be important to elucidate more specific biological mechanisms, and that high-quality epidemiologic studies are needed to provide more robust pathogen-specific evidence.

Regarding treatment exposure, we agree that systemic corticosteroids, immunosuppressants, biologics, and Janus kinase inhibitors may influence infection risk and complicate interpretation of infection–disease associations.^4^ It should be noted that our meta-analysis focused on infection as an exposure preceding incident disease diagnosis. In this context, the influence of treatment on the directionality of the association is inherently limited, as most included participants would not yet have received disease-specific therapies at the time of infection exposure. Nevertheless, residual confounding from preexisting treatments for comorbid conditions remains possible and cannot be fully excluded. More comprehensive reporting of treatment history, disease severity, and healthcare utilization in future studies would strengthen causal interpretation.

Despite these limitations, our study provides a synthesis to date of cohort evidence linking infection to subsequent risk of psoriasis and AD. We appreciate the comments by Islam et al, which highlight important methodological considerations and valuable directions for future research. Continued efforts to improve exposure characterization, phenotype definition, and reporting standards will further strengthen our understanding of the complex relationship between infection and inflammatory skin disease.

## Conflicts of interest

Xiao serves as Councilor of the International Psoriasis Council (IPC), member of the International Society of Atopic Dermatitis (ISAD), Associate Editor of the Journal of Investigative Dermatology (JID), and member of the Large Database Workgroup of the Journal of the American Academy of Dermatology (JAAD).
